# Consumption of Dairy Products and Cancer Risks

**DOI:** 10.2188/jea.17.38

**Published:** 2007-04-10

**Authors:** Masatoshi Matsumoto, Shizukiyo Ishikawa, Yosikazu Nakamura, Kazunori Kayaba, Eiji Kajii

**Affiliations:** 1 Division of Community and Family Medicine, Center for Community Medicine, Jichi Medical University.; 2 Department of Public Health, Jichi Medical University.; 3 Department of Nursing, School of Health and Social Services, Saitama Prefectural University.

**Keywords:** Dairy Products, Risk, Leukemia, Lymphoma, Cohort Studies

## Abstract

**BACKGROUND:**

Relationships between consumption of dairy products and death from various types of cancer are largely unknown.

**METHODS:**

Between April 1992 and July 1995, a baseline survey was conducted for 11,349 residents in 12 communities in Japan, which included collection of demographic data and a self-administered food-frequency questionnaire inquiring about three dairy products: milk, butter and yogurt. The subjects were followed prospectively until 2002. Causes of death were identified using death certificates. Hazard ratios (HRs) and their 95% confidence intervals (CIs) for each dairy product were calculated using Cox s proportional hazard models.

**RESULTS:**

Among eight common cancers, only deaths from hematopoietic neoplasm (n=14) were significantly associated with consumption of butter (HR=5.11, 95% Cl: 1.40-18.62), though they exhibited a nearly-significant association with milk consumption (HR=3.17, 95% Cl: 0.99-10.17), independent of age and sex. Consumption of milk and butter was significantly associated with non-lymphoma deaths (n=9) when adjusted for age and sex (HR=9.86, 95% Cl: 1.23-79.19 for milk: and HR=10.04, 95% Cl 2.39-42.18 for butter).

**CONCLUSION:**

The frequencies of butter consumption, and probably that of milk, were correlated with death from hematopoietic neoplasm, particularly from non-lymphomas.

Dairy products, such as milk, butter, and yogurt, are important components of the human diet. They are a source of fat, calcium, vitamin A, and many other essential elements for human health. Dairy products contribute 4% of the total energy intake consumed worldwide and 10% in North America and Europe.^1^ This proportion is increasing dramatically in some regions of the world, particularly in Asia. Between 1955 and 1995, for example, the consumption of dairy products increased 10-fold in Japan.^2^ Consequently, the role dairy products play in human health is becoming increasingly important.

Dairy products have been proposed as a possible risk factor for some types of cancer in ecologic and experimental studies, but their roles in the development of cancer have not been confirmed in case-control or large scale cohort studies.^3^^-^^11^ In particular, there are too few epidemiologic studies that have investigated the relationship between milk consumption and blood tumors,^12^^-^^14^ even though a possible relationship between bovine leukemia virus (BLV) and leukemia increasingly has been documented, at both the molecular and cellular level.^15^^-^^22^

Therefore, in this prospective cohort study, conducted across 12 communities in Japan, we investigated the relationships between the consumption of three different dairy products and death from various types of cancer.

## METHODS

### Baseline Survey

The Jichi Medical School (JMS) Cohort Study began in 1992. Its primary objective was to clarify the relationship between potential risk factors -- like life-style, socio-economic variables, serum lipids and other risk factors -- and certain health outcomes like stroke, cardiovascular disease, and cancer in 12 rural districts in Japan: Iwaizumi, Yamato, Takasu, Sakugi, Ainoshima, Akaike, Okawa, Hokudan, Kuze, Wara, Sakuma, and Tako.^23^ The baseline data of this cohort study were obtained between April 1992 and July 1995. If several sets of data were obtained for an identical subject during the period, the first set was used as base-line.

In Japan, mass screening for cardiovascular diseases has been conducted since 1982, in accordance with the Health and Medical Service for the Aged Act of 1981. Invitations to this mass screening were issued by local government offices in each community, and personal invitations were also sent to all potential subjects by mail. The subjects for the mass screening examinations were residents between the ages of 40 and 69 years in Iwaizumi, Tako, Kuze, Sakuma, Sakugi, Okawa, Ainoshima, and Akaike; and 30 years and older in Wara. Subjects for other age groups were included in Yamato, Takasu, and Hokudan. As a result, 12,490 subjects were eligible (4,913 males and 7,577 females) for all ages (19-93 years of age): 1,119 for Iwaizumi, 2,851 for Tako, 2,404 for Yamato, 450 for Kuze, 1,478 for Takasu, 1,371 for Wara, 306 for Sakuma, 1,129 for Hokudan, 394 for Sakugi, 214 for Okawa, 136 for Ainoshima and 638 for Akaike. The overall response rate among the 12 communities was 59.4%. Written informed consent to participate in the study was obtained individually for the respondents of mass screening. In this study, we excluded 793 subjects who had not answered any of the food questions concerning dairy products and 91 subjects who declined to be followed up. Thus, the final number of study subjects was 11,606.

To obtain baseline information using a uniform method, we established the central committee, which was composed of the chief medical officers from all of the participating districts, and this committee developed a detailed manual for data collection. Habitual food intake was assessed by means of a five-level scale intake-frequency questionnaire, which had been created based on a questionnaire used in previous studies,^24^^,^^25^ and which had been sanctioned by the central committee. The food-frequency questionnaire (FFQ) was employed for assessing the consumption of 30 food items including three dairy products: milk, butter, and yogurt. The questionnaire was administered once within three years of the baseline survey. On the FFQ, respondents rated each dairy product from one to five, relative to their frequency of intake: 1, "seldom"; 2, "1-2 times per month"; 3, "1-2 times per week"; 4, "3-4 times per week"; and 5, "almost everyday". No follow-up survey using the same FFQ or using dietary intake records was conducted in this study. A study which employed the same FFQ as ours (but included more food items than ours) reported that the correlation coefficients between FFQ estimates and estimates of actual consumption derived from 12-day diet records were 0.65 for milk, 0.58 for yogurt, 0.51 for butter, all of which were in the highest ten out of the 39 food items of the study.^25^ This means that the FFQ has a relatively high vilidity for consumption of the dairy products versus other food items, when examined in the Japanese population. Thus, we considered using the FFQ alone to be valid for assessing food habits.

### Follow-up

Subjects were followed until the end of 2002. Causes of death were identified using death certificates, which were collected at the respective local public health centers, with permission from the Ministry of General Affairs and the Ministry of Health, Labour and Welfare. The people who moved out of the communities during the observation period were followed until their date of emigration. Data on emigration of study subjects were obtained every year from their municipal governments.

### Statistical Analyses

Statistical analyses were carried out using SPSS® for Windows, version 11.5 (SPSS Inc, Japan). In order to evaluate the association between the consumption of each dairy product and death rates from various cancers, hazard ratios (HRs) were calculated, with 95% confidence intervals (CIs), using Cox's proportional hazard models. In this model, the influence of each dairy product on cancer death was adjusted for age and sex. For any cancer which demonstrated a significant relationship with the consumption of a dairy product, Cox's proportional hazard analyses were again conducted for further evaluation. For the analyses, consumption of each dairy product was recoded as a binary variable, divided into 'everyday (5)' and 'not everyday (1-4)' categories.

## RESULTS

All the demographic data are summarized in Table 1. Follow-up rate was 90.9%. Among participants, 60.8% were females and 39.2% were males. The average length of follow-up was 9.15 years. The average age of the subjects was 55.2 years, and the age distribution was from 18 to 90 years. The distribution of FFQ scores for dairy products is shown in Table 2. Note that 44.8% of the subjects drank milk almost everyday, while 6.5% ate butter and 5.6% ate yogurt almost everyday.

**Table 1.** Basic demographic data of the participants.

	n (%)	
Subjects at baseline	12490	( 100 )
Of those, subjects who were analyzable^*^	11606	( 92.9 )

Sex		
Male	4531	( 39.0 )
Female	7075	( 61.0 )

Average length of follow-up (year)	9.2	

Average age (year)	55.3	

Age distribution (year)		
18-29	242	( 2.1 )
30-39	838	( 7.2 )
40-49	2405	( 20.7 )
50-59	3081	( 26.5 )
60-69	4430	( 38.2 )
70-79	522	( 4.5 )
80-90	88	( 0.8 )

*: Subjects who were followed up and have completed any of the questionnaire relating to the consumption of dairy products.

**Table 2.** Distribution of subjects according to the five categories of dairy products.

Frequency	milk (%)	butter (%)	yogurt (%)
1: Seldom	2106 ( 18.1 )	4424 ( 38.1 )	5637 ( 48.6 )
2: 1-2 times/month	888 ( 7.7 )	2945 ( 25.4 )	2414 ( 20.8 )
3: 1-2 times/week	1592 ( 13.7 )	2348 ( 20.2 )	1899 ( 16.4 )
4: 3-4 times/week	1745 ( 15 )	1008 ( 8.7 )	887 ( 7.6 )
5: Almost everyday	5195 ( 44.8 )	759 ( 6.5 )	655 ( 5.6 )
Unavailable response	80 ( 0.7 )	122 ( 1.1 )	114 ( 1 )

Total	11606 ( 100 )	11606 ( 100 )	11606 ( 100 )

Table 3 shows the HRs with 95% CIs for each dairy product relative to each type of cancer death for which the absolute number of deaths was ten or more. The HRs were adjusted by age and sex. Among the eight common cancers, only hematopoietic neoplasm was significantly associated with the consumption of a dairy product (butter). In spite of the lack of significance, the association between milk consumption and hematopoietic neoplasm was close to significant (HR=3.17, 95% CI: 0.99-10.17).

**Table 3.** Associations between death from each type of cancer and consumption of dairy products: age and sex adjusted analysis.

Site of cancer	No of deaths	Type of dairy product	Hazard ratio (95% confidence interval)^*^		p
Colon	25	milk	1.26	( 0.57 - 2.78 )	0.56
		butter	0.64	( 0.09 - 4.70 )	0.66
		yogurt	1.28	( 0.30 - 5.48 )	0.74

Stomach	32	milk	0.70	( 0.34 - 1.43 )	0.40
		butter	1.58	( 0.48 - 5.20 )	0.45
		yogurt	0.47	( 0.06 - 3.46 )	0.46

Lung	56	milk	0.88	( 0.52 - 1.51 )	0.65
		butter	1.66	( 0.66 - 4.15 )	0.28
		yogurt	0.95	( 0.29 - 3.03 )	0.92

Liver	13	milk	0.83	( 0.27 - 2.54 )	0.74
		butter	NA^**^		
		yogurt	NA^**^		

Pancreas	13	milk	0.97	( 0.33 - 2.90 )	0.96
		butter	1.30	( 0.17 - 10.02 )	0.80
		yogurt	1.17	( 0.15 - 9.10 )	0.88

Bile duct	10	milk	0.70	( 0.20 - 2.47 )	0.57
		butter	1.68	( 0.21 - 13.32 )	0.62
		yogurt	2.77	( 0.58 - 13.30 )	0.20

Blood	14	milk	3.17	( 0.99 - 10.17 )	0.05
		butter	5.11	( 1.40 - 18.62 )	0.01
		yogurt	1.25	( 0.16 - 9.65 )	0.83

Others	92	milk	1.08	( 0.64 - 1.82 )	0.78
		butter	0.85	( 0.27 - 2.73 )	0.79
		yogurt	1.93	( 0.96 - 3.87 )	0.06

All cancers	255	milk	1.07	( 0.83 - 1.38 )	0.58
		butter	1.39	( 0.87 - 2.22 )	0.17
		yogurt	1.48	( 0.59 - 3.72 )	0.41

Consumption of each dairy product was analysed as a binary variable, subdivided into 'everyday (5)' and 'not everyday (1-4).'

* : Cox's proportional hazard model, adjusted by sex and age

**: Hazard ratio could not be calculated, because there was no case in the 'everyday (5)' category cell.

The deaths from hematopoietic neoplasm included five cases of malignant lymphoma, four cases of multiple myeloma, three of acute myelocytic leukemia, one of acute lymphocytic leukemia, and one of unclassified leukemia. The proportion of leukemia deaths relative to deaths from all causes was 0.76% (5/642), which was almost equivalent to its counterpart in the 2000 national database (0.76%).^26^ Because hematopoietic neoplasm comprises a heterogeneous mix of tumors, it was subcategorized into lymphomas and non-lymphomas for further analysis.

Table 4 shows the HR with 95% CI for the consumption of each dairy product relative to death from hematopoietic tumors. Because risks of hematopoietic tumors are scarcely known except for history of immune diseases and irradiation which are unavailable data in this study, we used age and sex as adjusting variables. Milk consumption was not associated with lymphoma deaths. In contrast, consumption of milk and consumption of butter were associated significantly with non-lymphoma tumor deaths. Yogurt was not associated with any type of hematopoietic tumor deaths.

**Table 4.** Consumption of dairy products and subclasses of hematopoietic tumors: age and sex adjusted analysis.

Type	Type of dairy product	Hazard ratio^*^ (95% confidence interval)	p
Lymphoma	milk	0.89 ( 0.15 - 5.40 )	0.90
	butter	NA^**^	
	yogurt	NA^**^	

Non-lymphoma	milk	9.86 ( 1.23 - 79.19 )	0.03
	butter	10.04 ( 2.39 - 42.18 )	0.02
	yogurt	1.96 ( 0.24 - 15.81 )	0.53

* : Cox's proportional hazard model, adjusted by age and sex

**: Hazard ratio could not be calculated, because there was no case in the 'everyday (5)' category cell.

## DISCUSSION

The results of the current study indicate that the frequency of butter consumption and probably that of milk consumption are correlated with death from hematopoietic neoplasm. The statistical significance of the relationship remains (butter) or even strengthened (milk) after deaths from lymphoma are excluded. This means that consumption of these two dairy products affects the overall rate of death from leukemia and multiple myeloma. As for death from other cancers, no significant association with dairy products was identified.

The relationship between the intake of dairy products and cancer has been a controversial issue for a long time. Positive correlations have been found between milk consumption and breast cancer, colon cancer, prostate cancer, and ovarian cancer in ecologic studies using international data.^3^^,^^4^ However, observational studies have not supported these findings. Various case-control studies, as well as cohort studies, have reported heterogeneous findings which were not necessarily supportive of an adverse effect of milk consumption on colon cancer.^6^^,^^7^ As for ovarian cancer, case-control studies have reported that the association between milk intake and ovarian cancer is inconclusive.^8^ Milk consumption has been reported to be a risk factor for prostate cancer in various case-control studies, but not in cohort studies.^9^^,^^10^ A cohort study which comprehensively examined the relationships between milk consumption and most cancers revealed that no cancer except malignant lymphoma was significantly correlated with milk consumption.^11^ The results of our study were in accordance with results from previous studies, except that milk consumption had an association with deaths from hematopoietic neoplasm excluding lymphoma.

The etiology of the most hematopoietic tumors largely remains unknown. The only well-established risk factors are immune system impairment and radiation.^27^^-^^28^ Exposure to some chemicals is another possible risk factor for leukemia. Cigarette smoking has been suggested as a risk factor for adult leukemia, but the estimates of relative risk are quite small.^29^ In short, these risk factors account for only a small proportion of the adult cases of hematopoietic tumors, and in most cases the causes for them are unknown.

As far as we know, two prospective studies have reported on an association between dairy consumption and hematological tumors. One cohort study has demonstrated a positive association between milk consumption and death from lymphoma,^11^ but not with death from leukemia or multiple myeloma. In contrast, the other study did not find any significant relationship between milk consumption and morbidity of lymphoma or leukemia.^12^ In case-control studies as well, one study argued for a relationship between non-Hodgkin's lymphoma and milk consumption,^13^ while another argued against it.^14^ These findings contradict the results of our study, which indicate a positive relationship between milk consumption and 'non-lymphoma' category of hematopoietic neoplasm. The reason for these incongruent results is unknown. Previous studies have never examined the association between hematopoietic neoplasm versus butter and yogurt.

Bovine leukemia virus (BLV) may be the factor which intervenes between the consumption of dairy products and the onset of human blood tumors. BLV is a horizontally-transmitted virus which causes the adult enzootic form of bovine leukemia, the most common fatal malignancy among dairy cattle. Approximately 1% to 5% of infected cattle develop leukemia, and the others are healthy carriers of the virus. The virus is in the same group as the human T-cell leukemia virus type 1 (HTLV-1), which is an oncovirus of human species.^15^ The infection rate of BLV in dairy cattle is known to be between 13% and 48% in the United States, 84% in Argentina, and 70% in Canada.^16^^-^^19^ Seventy percent of infected cows were reported to excrete BLV into their milk.^20^ Although the infectivity of BLV is inhibited by pasteurisation,^21^ it is unknown whether pasteurisation destroys the biological activity of the pro-viral BLV DNA in the infected cells of milk.^20^ In fact, a study from the US reported that antibodies reactive against BLV capsid antigen were detected in 74% of samples from the general population. This result does not necessarily mean that the people are infected with BLV, but indicate that a substantial percentage of people is exposed to antigens derived from BLV.^22^

The relationship between BLV and human leukemia has been supported by epidemiologic and experimental studies. Some case-control studies have reported that people engaging in dairy farming have a higher incidence of leukemia than the general population.^30^^-^^33^ In addition, a positive geographic correlation between BLV-infected cattle populations and human leukemia has been reported.^34^^,^^35^ However, there is no prospective study which shows any positive correlation between the intake of dairy products and human leukemia incidence. BLV in cow milk was reported to infect chimpanzees. Under experimental conditions, when six infant chimpanzees were fed infected cow's milk, two of them died as a result of leukemia within a year.^36^ In vitro, BLV infects cells of various origins, including human and simian cells.^37^

Neither the above-mentioned evidence nor the results of our study resolve the controversy surrounding BLV and human hematopoietic tumors. However, this combination of findings suggests that we should not take this issue lightly. The problem of BLV-infected cows and their effect on human health can have a great impact on public health policy, as well as an economic impact upon the dairy industry in many countries.^35^ Although the problem of BLV infected cows has not been taken seriously in most countries so far, Finland did tackle this issue as a national project and succeeded in eradicating BLV-infected cows from the country.^38^

Contrary to leukemia, there is a dearth of experimental or epidemiologic evidence indicating any association between BLV and human lymphoma (non-Hodgkin's or Hodgkin's lymphoma). That we identified a significant association between the consumption of dairy products and deaths from hematopoietic neoplasm, even after lymphoma deaths are excluded, supports the hypothesis that leukemia/myeloma, rather than lymphoma, is the effect of dairy product consumption. With respect to multiple myeloma, there also is no epidemiologic evidence supporting its association with dairy products. At a molecular level, however, BLV can infect human myeloma cells, as well as leukemia cells.^39^^,^^40^ Whether human multiple myeloma is associated with BLV infection has yet to be determined.

Four out of nine (44%) 'non-lymphoma' blood tumor deaths in our study were secondary to multiple myeloma. Although data are not shown, we conducted a further subgroup analysis separating myeloma from leukemia for the assessment of their individual association with dairy products. Multiple myeloma did not exhibit any significant associations (HR=3.61, 95% CI: 0.37-34.82 for milk; and HR=5.69, 95% CI: 0.59-55.06 for butter), whereas leukemia demonstrated a significant association with butter consumption (HR=16.32, 95% CI: 2.29-116.57); HR for milk could not be calculated because no case was in the 'not everyday' category of consumption. These results indicate that the significant association between 'non-lymphoma' blood tumor deaths and dairy product consumptions stems more from leukemia than from myeloma. Despite whether the correlations are statistically significant or not, however, all the confidence intervals of the subdivided blood tumors are so large that the results should be interpreted cautiously.

Admittedly, our study has some limitations. A particular problem inherent in epidemiologic studies on food and cancer pertains to the accuracy of assessments of diet content. Food is a difficult variable to investigate, because estimates of intake may not be accurate, relative to other quantifiable factors.^41^ Self-reported dietary habits are particularly susceptible to measurement error, because people just do not remember everything they eat. In short, the objectivity and reproducibility of the food data always are at issue. Another limitation is that the CIs for HR for each dairy product were wide because the number of hematopoietic neoplasm cases we observed was limited, which decreases confidence in the results. This especially must be considered because of the potential problem of multiple comparisons. We conducted multiple comparisons among eight tumors with three dairy products. Thus, it is possible that the statistically significant correlations observed with hematopoietic neoplasm occurred entirely by chance. We cannot deny this possibility. However, we consider the HRs for milk and butter relative to all hematopoietic tumors (3.17 for milk and 5.11 for butter) and non-lymphoma malignancies (9.86 for milk and 10.04 for butter) were too large to be haphazard. Nonetheless, further studies, with more cases and prolonged observation periods, are needed to confirm our findings.
